# Super-resolution microscopy reveals decondensed chromatin structure at transcription sites

**DOI:** 10.1038/srep04477

**Published:** 2014-03-26

**Authors:** Yejun Wang, Shovamayee Maharana, Michelle D. Wang, G. V. Shivashankar

**Affiliations:** 1 Mechanobiology Institute, Singapore; 2 Department of Biological Sciences, National University of Singapore, Singapore; 3 Laboratory of Atomic and Solid State Physics, Department of Physics; 4 Howard Hughes Medical Institute Cornell University, Ithaca, New York 14853, USA

## Abstract

Remodeling of the local chromatin structure is essential for the regulation of gene expression. While a number of biochemical and bioimaging experiments suggest decondensed chromatin structures are associated with transcription, a direct visualization of DNA and transcriptionally active RNA polymerase II (RNA pol II) at super-resolution is still lacking. Here we investigate the structure of chromatin isolated from HeLa cells using binding activatable localization microscopy (BALM). The sample preparation method preserved the structural integrity of chromatin. Interestingly, BALM imaging of the chromatin spreads revealed the presence of decondensed chromatin as gap structures along the spreads. These gaps were enriched with phosphorylated S5 RNA pol II, and were sensitive to the cellular transcriptional state. Taken together, we could visualize the decondensed chromatin regions together with active RNA pol II for the first time using super-resolution microscopy.

Eukaryotic DNA is packaged, together with histones and non-histone proteins, into chromatin fibers^1^,^2^. The fundamental unit of this fiber is the nucleosome^3^, which consists of ~150 base pairs of DNA wrapped 1.6 times around an octamer of core histones (H2A, H2B, H3, H4) and sealed with a single linker histone (H1) molecule that is bound closely to the core particle dyad^4^,^5^. In interphase cells, chromatin fibers are further packed into higher order structures composed of euchromatin and heterochromatin^6^. Euchromatin is mostly comprised of active genes and gene-rich regions, while repressive DNA is usually heterochromatinized^7^. The various levels of DNA packaging are mediated by a number of post-translational modifications on both core and linker histones^8^,^9^.

Modulation of the chromatin structure at promoter sites is required for eukaryotic transcription, and this occurs in a highly regulated manner^10^,^11^. A number of studies have explored the structure of chromatin using electron microscopy^12^,^13^, while X-ray crystallography studies have described the structure of the nucleosome and the DNA-protein complexes at a resolution of Ångströms^14^,^15^,^16^,^17^,^18^. Other optical microscopy methods have also revealed chromosome territories at a resolution above hundreds of nanometers^19^,^20^. However, the packaging of DNA beyond 10 nm at both euchromatin and heterochromatin regions is largely unclear. This is due to restrictions with electron microscopy labeling methods and the limited resolution associated with conventional light microscopy. Recently, a number of ‘super-resolution’ strategies were developed that circumvent the usual optical resolution limits^11^,^21^,^22^,^23^,^24^. One simple yet powerful method that is becoming more widely adapted is ‘single-molecule localization microscopy’. This method, which can obtain a lateral spatial resolution of ~20 nm^25^,^26^,^27^,^28^, involves the repeated imaging of sparse stochastic subsets of fluorophores in a single sample. The position of each fluorophore is determined by finding the center of their point spread function, and this information is used to construct a super-resolution image.

In this paper, we visualized the chromatin structure on open chromatin spreads prepared from interphase cells, using both total internal reflection fluorescence microscopy (TIRFM) and binding activatable localization microscopy (BALM), which was recently developed by Schoen, I., *et al.*^29^. Co-localization of DNA with histone proteins (e.g. H1, H2B), was observed using the TIRFM technique, indicating chromatin fibers in the open spreads were structurally intact after an appropriate nuclear expansion time. Through the use of the BALM technique, a substantial enhancement in resolution of chromatin fibers was attained compared to the TIRFM technique. The most common type of fiber observed via BALM had a width of 150 ± 45 nm (mean ± SD), whilst those observed with TIRFM had a width of 450 ± 30 nm. Structural changes of chromatin in actively transcribing (serum (+)) versus quiescent (serum (−)) states were also detected using BALM. In the actively transcribing state, chromatin fibers were less compact and featured more gap structures, which were defined as decondensed regions having a length of 388 ± 170 nm (mean ± SD) and a width of 60 ± 25 nm (mean ± SD). To further check the transcriptional activity of these gap structures, we immunostained phosphorylated S5 RNA pol II. Active RNA pol II is phosphorylated at the 5^th^ serine in the heptad YSPTSPS of the C-terminal domain. This active RNA pol II is recruited to gene promoters during transcription initiation. Colocalization of RNA pol II with gap structures implies that these gap structures may be transcriptionally active. Consistent with this, when cell quiescence was induced via serum starvation, the number of gap structures, as well as RNA pol II signals in those regions, decreased. In conclusion, we show that super-resolution microscopy revealed decondensed chromatin regions with transcriptionally active RNA pol II.

## Results

### Characterization of chromatin fibers

**Figure 1:**
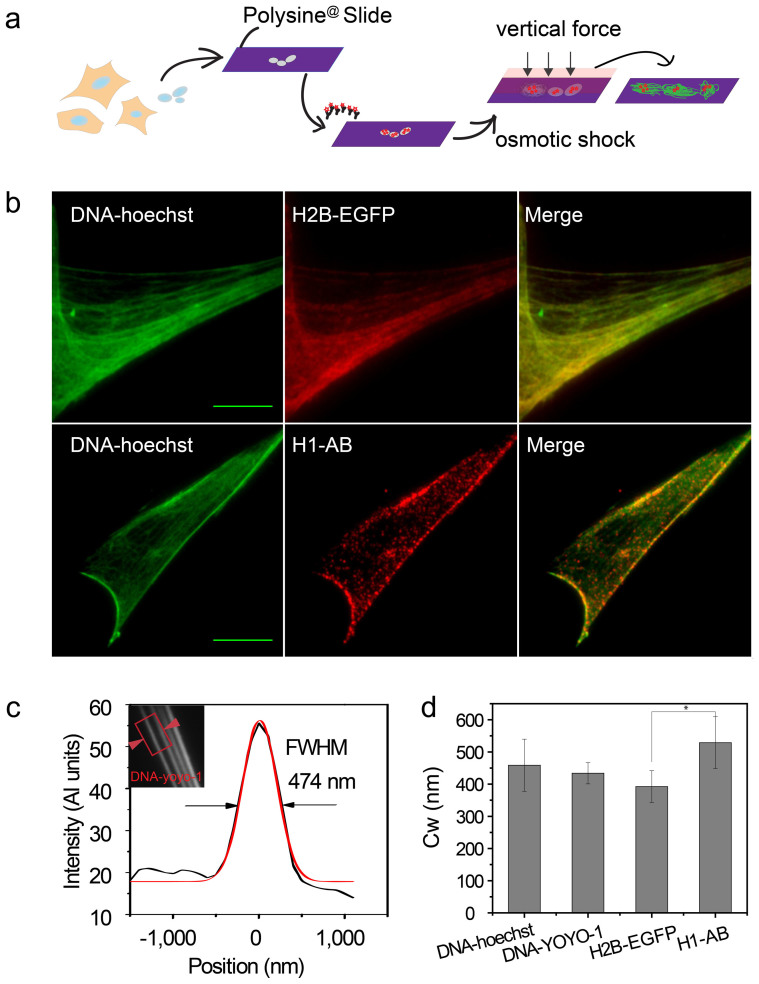
Functionality of chromatin fibers | (a) Schematic of chromatin fiber preparation (b) Representative TIRFM images of the colocalization of DNA and histone proteins on chromatin fibers. Scale bar: 10 μm. (c) Line profile for determination of FWHM (representing chromatin width (Cw) in the inset red box) (d) Bar graph showing chromatin width (Cw) in four labeling ways: DNA stained with hoechst (DNA-hoechst), DNA stained with YOYO-1 (DNA-YOYO-1), H2B tagged with EGFP (H2B-EGFP), and H1 immunolabeled with antibodies (H1-AB) (n ≥ 20, all the ‘n’ in the following text refers to the number of fibers) (*P < 0.05; Student's t-test).

Isolated nuclei from HeLa cells were seeded on polylysine-coated glass microscopy slides and swollen using deionized (DI) water for 10–30 minutes. Force, exerted by moderate tapping through a coverslip, resulted in chromatin being spread into strands, which were subsequently visualized by total internal reflection fluorescence microscopy (TIRFM) (Fig. 1a, b). This sample preparation method produced long chromatin fibers on the coverslip with the preservation of large-scale chromatin structures. The large-scale morphology of the spread chromatin ranged from long cable-like structures, which were up to several hundred microns long, to an array of shorter fibers (Supplementary Fig. S1a–d).

**Figure 2:**
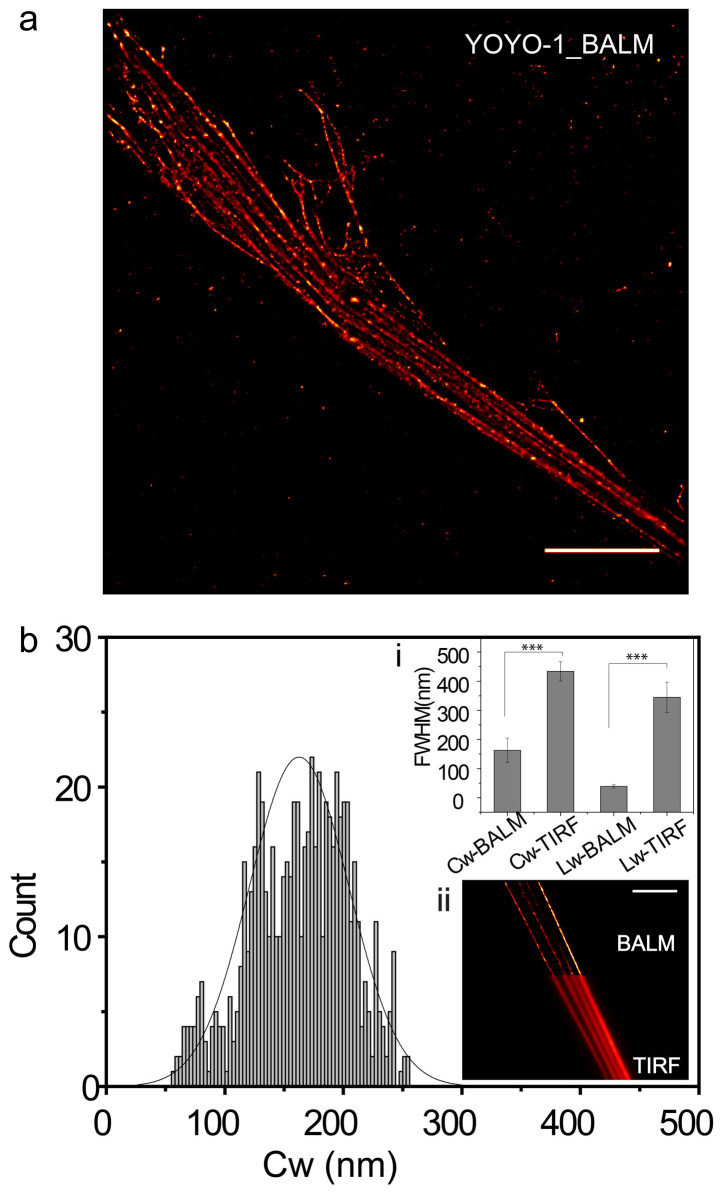
BALM images of chromatin fibers | (a) A representative BALM image of chromatin fibers stained with YOYO-1. Scale bar: 10 μm. (b) Histogram showing the distribution of chromatin width (Cw). Inset i shows the chromatin width (Cw) and λDNA width (Lw) in BALM image and TIRF image (n ≥ 20) (***P < 0.001; Student's t-test). Insect ii shows a combination of BALM and TIRF images of the same sample. Scale bar: 10 μm.

**Figure 3:**
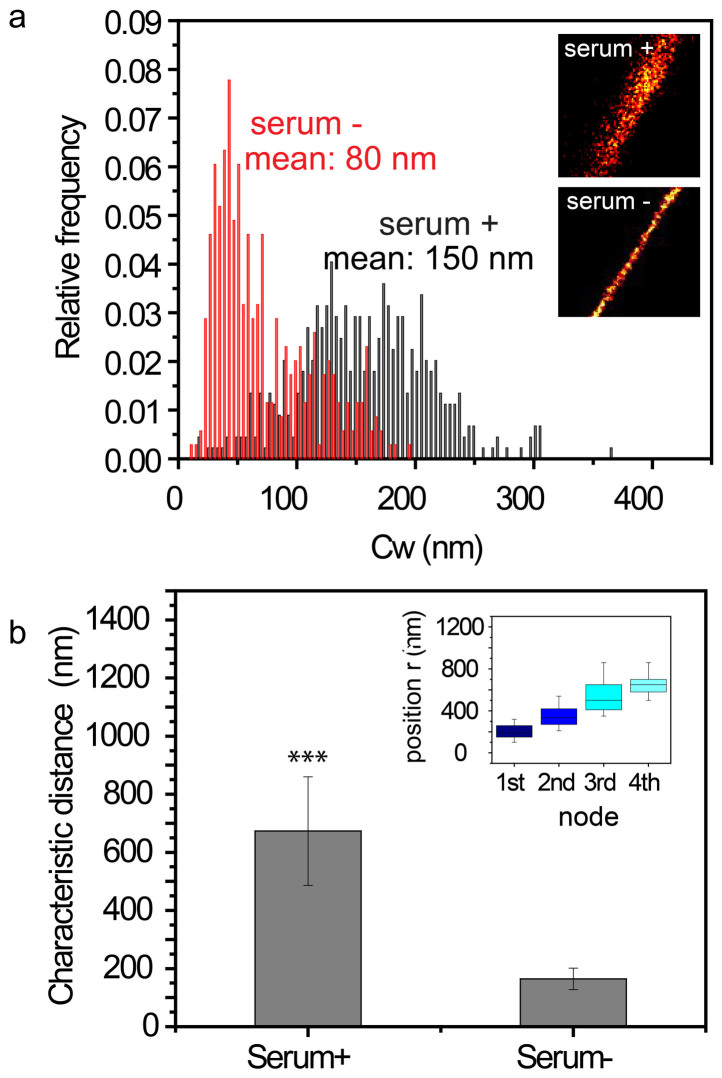
BALM detects different compaction states of chromatin in serum +/− conditions. | (a) Normalized histogram of the chromatin width (Cw) in serum +/− conditions. Insets are the representative BALM images of serum +/− chromatin. (b)Bar graph shows the characteristic distance between two nodes of chromatin fibers (n ≥ 15) in serum +/− conditions from spatial correlation analysis (***P < 0.001; Student's t-test). Insets: Box graph shows the periodicity of nodes.

Histone protein H2B, and the highly dynamic H1^30^, co-localized with DNA in the chromatin spreads (Fig. 1b, supplementary Fig. S1e–h). Chromatin fiber width (CW) was quantified by measuring the full width at half maximum (FWHM) on the line (with a height of 10 pixels) intensity plot across the fiber (Fig. 1c, inset)^29^. The final width of the chromatin was calculated by averaging measurements taken at multiple positions on multiple fibers (Fig. 2b, Fig. 3a, Supplementary Fig. 3d). Four different labeling methods were used in the visualization and quantification of chromatin width. These were DNA labeled with Hoechst (DNA-Hoechst) (460 ± 80 nm), DNA labeled with YOYO-1 (DNA-YOYO-1) (450 ± 30 nm), H2B tagged with EGFP (H2B-EGFP) (400 ± 50 nm), and H1 stained with antibodies (H1-AB) (500 ± 80 nm). No significant difference in chromatin width existed when comparing the first three methods, however, compared to H2B-EGFP, H1 stained with antibodies (H1-AB) resulted in chromatin fibers that were 25% thicker (Fig. 1d). The extra width observed using H1-AB may be the consequence of two factors: firstly, H1 is the linker histone on the surface of the nucleosome, while H2B is a core histone located in the center of nucleosome. Secondly, H1 was labeled by primary and secondary antibodies (approximately 150 kDa), whose size cannot be ignored, while H2B was tagged with a small GFP protein (approximately 27 kDa). Also, compared to other fibers, the fibers labeled with H1-AB are more discontinuous, and this could be because of the highly dynamic nature of linker histone H1. Supplementary Table S1 describes the probe binding sites for the four labeling methods.

Various durations of incubation with DI water resulted in different degrees of nuclear expansion. A short incubation time (<1 min) resulted in poor spreading whilst a long incubation time (>1 hour) produced well-expanded spreads (Supplementary Fig. S2a). Statistical analysis showed that after a longer incubation period (>1 hour) there was no significant difference in the width of the spread fibers (Supplementary Fig. S2b). However, the histone protein density, as well as RNA pol II density, decreased approximately 30% with the longer incubation (Supplementary Fig. S2c). To obtain good spreading, and retain the maximum number of DNA binding proteins, the nuclei were expanded for 10–30 min. During this time the loss of linker histone H1 was less than 20%, and no significant loss of RNA pol II was observed when compared to a shorter expansion time (time < 1 min) (Supplementary Fig. S2c).

### Enhancing the resolution of chromatin using BALM

Binding activatable localization microscopy (BALM) was used to yield further insights into the organization of chromatin fibers. This super-resolution technique was developed by Schoen, I., *et al.*^29^ in 2011. It resembles photoactivatable localization microscopy (PALM)^25^ and stochastic optical reconstruction microscopy (STORM)^23^, both of which are based on the detection of single-molecule, and provide single-molecule sensitivity with a spatial resolution of tens of nanometers. Cycles of stochastic switching, detection, and localization of single molecules on a TIRFM microscope were used to reconstruct super-resolution images (*Materials and Methods*). YOYO-1, a DNA intercalating dye that fluoresces around 800–1000 times more upon binding to DNA, has been reported to be a good marker for STORM imaging of DNA in a reducing buffer^31^. However, due to photobleaching during the progressive imaging process employed by STORM, the labeling density of YOYO-1 was found to be too low, and yielded a lower density of localization events on the chromatin fibers, as well as a loss of detail for many structures (Supplementary Fig. S5a). This problem was overcome by Schoen, I., *et al.*, and YOYO-1's labeling density improved, when its property of enhanced fluorescence following DNA binding was exploited by providing dynamic binding conditions. This gave rise to binding activatable localization microscopy (BALM) and using this imaging method, we were able to obtain more detailed images of chromatin fiber structures (Supplementary Fig. S5b).

To standardize the labeling regime and the imaging conditions, λ DNA was combed onto a positively charged (3-Aminopropyl) triethoxysilane (APTES) coated coverslip (*Materials Methods*, Supplementary Fig. S3a). YOYO-1 was added to the reducing-oxidizing system (ROXS buffer) (*Materials Methods*) and images were captured at a rate of 20 Hz. In solution, the YOYO-1 molecules remained dark until binding to DNA, at which point they became bright. This resulted in low background signals. Accumulated images of individual fluorophores localizing to the DNA allowed for the optical reconstruction of stretched double-stranded DNA (dsDNA) molecules (Supplementary Fig. S3b) with a FWHM, which is a measure of the lambda DNA width (Lw), of 30 ± 9 nm (mean ± SD) (Supplementary Fig. S3c). The resolution was defined by the finest fiber width that could be detected in BALM, which was ~20 nm (Supplementary Fig. S3 b, c insets). Since ROXS enhanced both the binding and disassociation rates of YOYO-1^29^, DNA was continuously bound and unbound by dye molecules from the solution. As a result, the number of localization events remained high (Supplementary Fig. S4) even in the later frames of acquisition. This ensured a uniform reconstruction of the λDNA structure was obtained (Supplementary Fig. S3b, inset). To get a well separated single molecule of λDNA on the coverslip, the λDNA stock was diluted to a concentration of 1 μg/ml, and most of the molecules that were selected for quantification possessed a length of ~20 μm, which is close to the predicted size of full-length λDNA.

**Figure 4:**
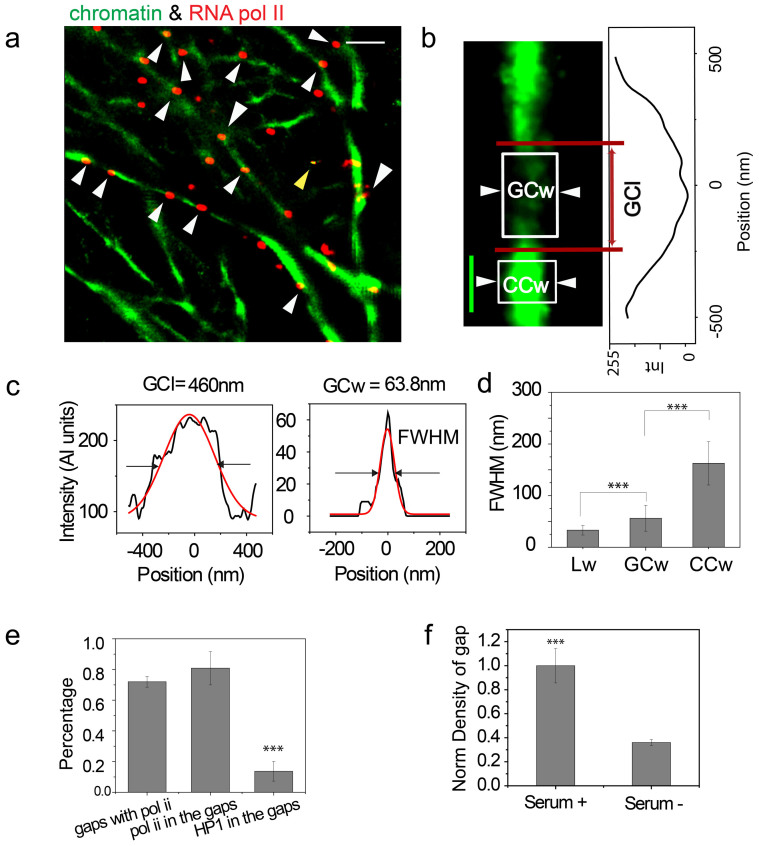
BALM can detect the transcriptionally active regions|(a) Dual- color BALM image of chromatin (green) and RNA pol II (red). Scale bar: 1 μm. White arrows indicate the RNA pol II signals, which are in the gap structures. The yellow arrow indicates the tetraspeck beads. (b) A representative zoomed in BALM image of gap structure with an intensity line plot along the structure. Scale bar: 200 nm. (c) Representative line profile for the gap chromatin length (GCl) and gap chromatin width (GCw) denoted by the red lines and white boxes in (b). (d) Box plot of the λDNA widh (Lw) (n = 50), gap chromatin width (GCw) (n = 50), and condensed chromatin width (CCw) (n = 50) (***P < 0.001; Student's t-test). (e) Bar graph showing the percentage of gaps with RNA pol II, the percentage of RNA pol II sitting in gaps, and the percentage of HP1α sitting in gaps (n = 20) (***P < 0.001; Student's t-test). (f) Bar graph showing the normalized density of gap structures along 10-μm fibers in serum +/− conditions (n = 30) (***P < 0.001; Student's t-test).

After using well-studied λDNA as a control to characterize the super-resolution imaging technique, we applied similar imaging conditions to visualize chromatin spreads. The reconstructed image of chromatin fibers with a width of 150 ± 45 nm (mean ± SD) showed dramatic enhancement in resolution when compared with diffraction-limited TIRFM image of chromatin fibers that have a width of 450 ± 30 nm (mean ± SD) (Fig. 2a, b, inset i, ii). Overall, the observed chromatin fibers ranged from less than 100 nm to ~400 nm (Fig. 2, Supplementary Fig. S5b, c). Gap structures were also revealed along the chromatin fibers following this detailed examination (Fig. 4a, b).

### Serum starvation induced chromatin condensation

Next, we tested if BALM could detect structural changes in chromatin induced by transcriptional quiescence. Cells were switched to a quiescent state by withdrawing serum from their growth medium for 36 hrs^32^,^33^,^34^. Chromatin spreads were subsequently obtained from these cells. Under serum withdrawal (serum (−)) conditions, the width of the observed chromatin fibers was 80 ± 40 nm (mean ± SD), which was substantially thinner than those observed under serum (+) conditions, which averaged 150 ± 45 nm (mean ± SD) (Fig. 3a). From the BALM images, serum (−) chromatin fibers had a higher photon density (Fig. 3a insets), which was caused by the higher DNA labeling density in serum (−) fibers. After filtering out low-level noise signals, distinct punctate structures (nodes) along fibers were observed (Supplementary Fig. S6a,c). Additionally, spatial correlation analysis (*Method and Materials*) showed smaller intervals (164 ± 37 nm (mean ± SD)) between two punctate structures in serum (−) chromatin fibers. This was compared to serum (+) chromatin fibers of the same length where the distance between two punctate structures was 673 ± 187 nm (mean ± SD) (Fig. 3b, Supplementary Fig. S6a–d).

The structural changes of chromatin at actively transcribing and quiescent states are important for the function and localization of transcriptional machinery. Because of this we next investigated regions of chromatin that are enriched with transcriptional machinery. The colocalization of chromatin and RNA pol II in chromatin spreads prepared with and without serum conditions were also compared.

### BALM detects transcriptional regions on chromatin fibers

Transcriptionally active RNA pol II (phospho S5CTD) was immunolabeled in isolated nuclei and this was followed by chromatin spreading. Chromatin fibers were labeled with YOYO-1. RNA pol II was labeled with a primary antibody (anti-RNA polymerase II CTD repeat YSPTSPS (phosphor S5), ab5131) and a secondary antibody conjugated with Alexa 647. Super-resolution images of RNA pol II were taken via direct STORM (dSTORM) by first increasing laser power to 100% and then decreasing to 2% for imaging. RNA pol II signals were found to be enriched in gap structures. These were characterized by regions of low YOYO-1 fluorescence intensity (Fig. 4a, Supplementary Fig. S10c). To quantitatively assess the correlation between RNA pol II and gap structures, BALM data sets, which were obtained from visualizing chromatin fibers, and dSTORM data sets, which were obtained from visualizing RNA pol II, were post-processed. This involved reconstructing ten thousand diffraction-limited images and subsequently retaining points with a localization precision of <20 nm in both data sets. The final images were constructed by fitting a Gaussian function (*Materials and Methods*) to each selected point in the images and merging them together. Line intensity profiles were plotted along fibers in the final image, and regions with a mean intensity at least two times lower than that of the neighboring region was defined as a gap structure (Figure 4b). Reconstruction of super resolution images from a different number of acquisition frames ruled out the possibility that gap structures were an imaging artifact (Supplementary Fig. S7). Gap structures were characterized by measuring their length (gap chromatin length: GCl) and width (gap chromatin width: GCw, Fig. 4b, c) and were found to be 388 ± 170 nm (mean ± SD) and 60 ± 25 nm (mean ± SD) respectively (Fig. 4d).

RNA pol II signals were considered to co-localize with chromatin fibers if the distance between their center point and the center of the fiber cross-section (D_p2c_) was within 20 nm (Supplementary Fig. S8). This distance also represented the upper limit of the localization precision in our experiments (Supplementary Fig. S3c inset). Similar post-processing was carried out when analyzing the localization of the transcription factor Serum Responsive Factor (SRF) on chromatin fibers. This transcription factor was also enriched in gap structures (Supplementary Fig. S9a). To statistically quantify the correlation between RNA pol II and the gap structures, we analyzed one hundred gap structures, which were chosen randomly from dual color images of RNA pol II and chromatin fibers. RNA pol II was found to be co-localized with ~70% of the gap structures analyzed (Fig. 4e). Similar analysis of one hundred randomly chosen RNA pol II signals showed 75% to be co-localized with gap structures (Fig. 4e). In contrast to this observation, only ~10% of the heterochromatin protein 1α (HP1α) signals were found to co-localize with gap structures (Fig. 4e).

The transcriptional relevance of gap structures was further tested by inducing transcriptional repression. This was achieved by withdrawing serum from culture medium. In serum (−) conditions, the normalized density of gap structures along 10-μm chromatin fibers was less than half of that measured under serum (+) conditions (Fig. 4f). Concomitantly there was a decrease in RNA pol II number, as well as SRF co-localization, on chromatin fibers (Supplementary Fig. S9).

## Discussion

The eukaryotic nucleus is an organelle that is densely packed with DNA and proteins. This density makes visualizing the local chromatin structure, as well as its interactions with functionally relevant proteins such as RNA pol II, particularly difficult. In this study we overcame these difficulties by swelling nuclei and preparing chromatin spreads via a technique that does not disrupt chromatin architecture, as evidenced by the retention of highly dynamic linker histones, as well as the core histones on the chromatin.

By combining open chromatin spreads with BALM, we have generated a robust yet simple strategy for visualizing the structure of active chromatin with a spatial resolution of 20 nm. This approach allows for the detection of structural changes in chromatin, specifically in fiber width, or in the characteristic distances between two punctate structures. This detection ultimately arises from the spatial analysis of chromatin fibers and can identify changes when the chromatin is in either an actively transcribing, or quiescent, state. By applying this technique, we noted that fiber width dropped from 150 ± 45 nm (mean ± SD) in the actively transcribing state to 80 ± 40 nm (mean ± SD) in the quiescent state. The reduced distance between two punctate structures reflected a higher density of punctate structures in the quiescent state than in the actively transcribing state.

These data implied that in a serum (−) condition, or quiescent state, chromatin fibers were more tightly packed together. This was contrasted by chromatin fibers in a serum (+) condition, or actively transcribing state, which tend to be loosely entangled with each other and form a relatively thick structure (Supplementary Fig. S6e). Our result is consistent with existing evidence that showed, using Hoechst staining, that serum starvation induces the formation of a more condensed chromatin state^35^.

Importantly, the compact organization of serum-starved chromatin fibers may be disrupted if expansion is permitted over an extended preparation time (>1 hrs). When this was explored in our study, a significant increase in chromatin width, and a significant decrease in H1 density became apparent (Supplementary Fig. S11). However, the density of RNA pol II did not decrease significantly and this was because of a reduced level of RNA pol II in the serum starved chromatin (Supplementary Fig. S9, S11). With each of these factors in mind, a 10–30 min expansion time for chromatin fiber preparation was used in all the experiments presented in this study. This allowed us to rule out artifacts arising from longer time expansion.

The super-resolution microscopy technique, BALM, provided a direct snapshot of previously unobserved gap structures on the chromatin fiber. These images, together with images captured on a dSTORM system, further revealed a correlation between gap structures and RNA pol II. ~75% of transcriptionally active RNA pol II was co-localized with gap structures, while only ~10% of the heterochromatin protein 1α (HP1α) was found in gap structures. A decrease in the transcriptional activity of cells, which was induced by withdrawing serum from their growth media, leads to a decrease in the number of gap structures, as well as RNA pol II and SRF punctae. We propose that gap structures are likely associated with transcription sites, and although we have not directly visualized transcription occurring at these sites in this work, this will be the subject of future studies. At present however, and despite the BALM technique providing a relatively higher resolution than other imaging techniques, limitations remain that prevent the resolution of single gene promoters. Our super-resolution microscopy based observations, do however show decondensed chromatin regions that are possibly composed of several genes, and are associated with active RNA pol II in mammalian cells.

## Methods

### Cell culture and serum starvation assay

Wild type HeLa cells and HeLa cells stably transfected with fusion plasmid for core histone H2B tagged with EGFP^36^ were cultured in Dulbecco's modified Eagle's medium (DMEM, Gibco, New York, USA) supplemented with 10% fetal bovine serum (FBS, Gibco) at 37°C in 5% CO_2_. To subject cells to serum starvation, cells were cultured in DMEM without FBS at 37°C in 5% CO_2_ for 36 hours.

### Nuclei isolation and chromatin spreads

HeLa cells were suspended in Phosphate Buffered Saline (PBS) after a brief treatment with Trypsin (Gibco). Cells were collected by centrifugation at 200×g and re-suspended in cell rupturing buffer TM2 containing 2 mM MgCl_2_, 10 mM Tris-HCl (pH 7.4), 5 mM PMSF (Sigma, USA) with 1% Triton X-100 for 4–5 minutes at 4°C. The nuclei were separated as pellet from the ruptured cytoplasm by centrifugation for 2 minutes at 400×g. The pelleted nuclei were separated from each other by rigorous tapping and stored in 1× PBS containing 1× protease inhibitor cocktail (Roche, Germany)^37^. Nuclei were allowed to settle on polysine-coated slides (MENZEL-GLASER Polysine® J2800AMNZ, Thermo Scientific, Germany) for 30 minutes by confining them in PDMS (DOW CORNING CORPORATION, USA) wells. Attached nuclei were swollen with deionized (DI) water and burst under a moderate pressure exerted through an 18 × 18 mm coverslip that was cleaned in detergent with ultrasonication for 30 min. The coverslip was sealed with appropriate imaging buffer (described later) on the slide and then subjected to imaging. All the experiments were performed in triplicate.

### Labeling of chromatin fibers and transcriptionally related proteins

After rupturing the nucleus of wild type HeLa cells, the DNA, which existed as chromatin fibers prepared as described above, was labeled with either 1 μg/ml Hoechst 33258 (Sigma-Aldrich, USA) or 100 ng/ml of YOYO-1 (Invitrogen, USA) diluted in freshly made ROXS buffer (50 mM Tris-HCl, 50 mM NaCl, 1 mM EDTA, 1 mM Methyl viologen (Aldrich, USA), 10 mM L-Ascorbic acid (Sigma, USA), pH7.5) for 5 min and mounted in ROXS buffer^29^ for imaging. For H2B-EGFP transfected HeLa cells, chromatin fibers were visualized in reducing buffer: 10 mM PBS (pH 7.4), 0.5 mg/ml glucose oxidase (Sigma), 40 μg/ml catalase (Sigma), 10% w/v glucose (Fischer Scientific), and 50 mM β-mercaptoethylamine (MEA, Fluka).

DNA-associated proteins were labeled with antibodies in isolated nuclei before the swelling process. For immunolabeling, isolated nuclei were incubated in blocking reagent (1% BSA in PBS), followed by primary antibody and secondary antibodies diluted in blocking reagent, each for ~30 min at room temperature. Linker histone H1 (Upstate 05-457, Merck Millipore), transcriptionally active CTD phosphorylated RNA pol II (ab5131, Abcam, UK) and Serum Response Factor, SRF (sc-25290, Santa Cruz biotechnology, USA) were immunolabeled on chromatin fibers.

### λDNA stretching

Pre cleaned 22 × 22 mm coverslips were rendered positively charged by coating with (3-Aminopropyl) triethoxysilane (APTES, Sigma-Aldrich, USA). Then, 1 μg/ml of λDNA (BioLab, New England) stained with YOYO-1 (at a dye/bp ratio of 1/150) was added onto the coverslips and incubated for ~30 min, allowing attachment of λDNA via one or more sites on the coverslip. The stretching of λDNA was achieved through capillary effect created by the rapid absorption of the buffer by tissue paper followed by the force caused by the surface tension of the receding liquid surface^38^.

### Super-resolution imaging

Super-resolution imaging was performed on a Zeiss Elyra P.1 microscope, equipped with an oil-immersion objective (alpha “Plan-Apochromat” 100X/1, 46 Oil DIC) and Total internal fluorescence (TIRFM) illumination. TIRFM illumination was achieved by using lasers with motorized TIRFM angle adjustment. The resulting illuminated area was 51.1 × 51.1 μm (with alpha “Plan-Apochromat” 100×/1.46 Oil DIC, full chip recording). Excitation was provided by a 488 nm laser line (100 mW) with AOTF-based intensity control. Emitted fluorescence was collected by the same objective and captured by an Andor iXon 897 back-thinned EMCCD camera. Integration time per frame was 50 ms at full laser power. Typically 10,000 frames were collected, which corresponded to measurement duration of ~10 min. XY drift and alignment differences between different channels were corrected by localizing 0.2-μm Tetraspeck beads (Invitrogen, USA) immobilized on the sample coverslip.

### Super-resolution data analysis

Raw data was processed using Zeiss Zen software to detect single-molecule events above background noise. A Gaussian filter and a Laplace filter were applied to every event of single molecule fluorescence in each frame of the raw image to reduce noise and enhance the detection of events. The image mean (M) and standard deviation (S) were then computed. Single-molecule events were defined when the peak intensity (I) satisfies: 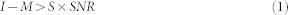
Where, SNR is a user-definable signal-to-noise ratio. The area to be analyzed around each event was typically set to 9 pixels. Events with overlapping PSFs were kept in order to localize the Tetraspeck beads for alignment. Gaussian fit was chosen as the method to calculate the center of detected PSFs. After reconstruction, a super-resolution image and a table containing the x-y coordinates of all the single-molecule events (and other details, notably the precision of each localization) were obtained. A typical super-resolution acquisition of YOYO-1-labeled chromatin contained from one hundred million to several billion total detected events. In the post-processing step, events which were above the 20 nm localization limit were discarded. A super-resolution (SR) image was generated by fitting each event with the Gaussian function, and binning the number of localizations with a bin size of 10 nm. The exported SR images were then processed in MATLAB and ImageJ and the morphological features of the spread were established.

### Spatial correlation analysis

A high-pass filter was applied in the Fourier domain of the reconstructed super-resolution image of chromatin fibers. This resulted in an image with only periodic node structures along a fiber, which originally existed together with other random structures in chromatin. A pixel-wise autocorrelation analysis was then carried out to determine the compaction of chromatin structures. To obtain characteristic length scales, the starting point of the fiber was set zero, each mean intensity value along the fiber was used as a signal to calculate the autocorrelation function 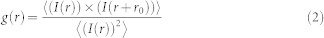
Where *I*(*r*) is the mean intensity value at position *r*, and *r*_0_ is the step moving along the fiber. The averaged autocorrelation *g*(*r*) was obtained from fibers with the length of 2 μm (n ≥ 15). All data analysis was carried out using LabVIEW 6.1 (National Instruments) and graphs were plotted in Origin 8.0 (OriginLab).

## Author Contributions

Y.W., S.M., M.W. and G.V.S. designed experiments. Y.W. and S.M. performed experiments. Y.W., S.M., M.W. and G.V.S. wrote the main manuscript text. All authors reviewed the manuscript.

## Supplementary Material

Supplementary Information Super-resolution microscopy reveals decondensed chromatin structure at transcription sites
