# Supplementary material for: Super-resolution microscopy reveals decondensed chromatin structure at transcription sites
Source: Sci Rep. 2014 Mar 26;4:4477. doi: 10.1038/srep04477 (PMC3966049; doi:10.1038/srep04477)
Supplement: Supplementary Information — Super-resolution microscopy reveals decondensed chromatin structure at transcription sites [file srep04477-s1.pdf]

Title:

Super-resolution microscopy reveals decondensed chromatin structure at transcription sites

Author Affiliations:

Yejun Wang[1], Shovamayee Maharana[1], Michelle D. Wang[3,4], & G.V. Shivashankar[1,2,*]

[1]Mechanobiology Institute, Singapore

[2]Department of Biological Sciences, National University of Singapore, Singapore

[3]Laboratory of Atomic and Solid State Physics, Department of Physics

[4]Howard Hughes Medical Institute Cornell University, Ithaca, New York 14853, USA

Corresponding Author:

G.V. Shivashankar, Mechanobiology Institute, National University of Singapore, T-Lab #05-01, 5A Engineering Drive 1, Singapore 117411.

Email: shiva.gvs@gmail.com

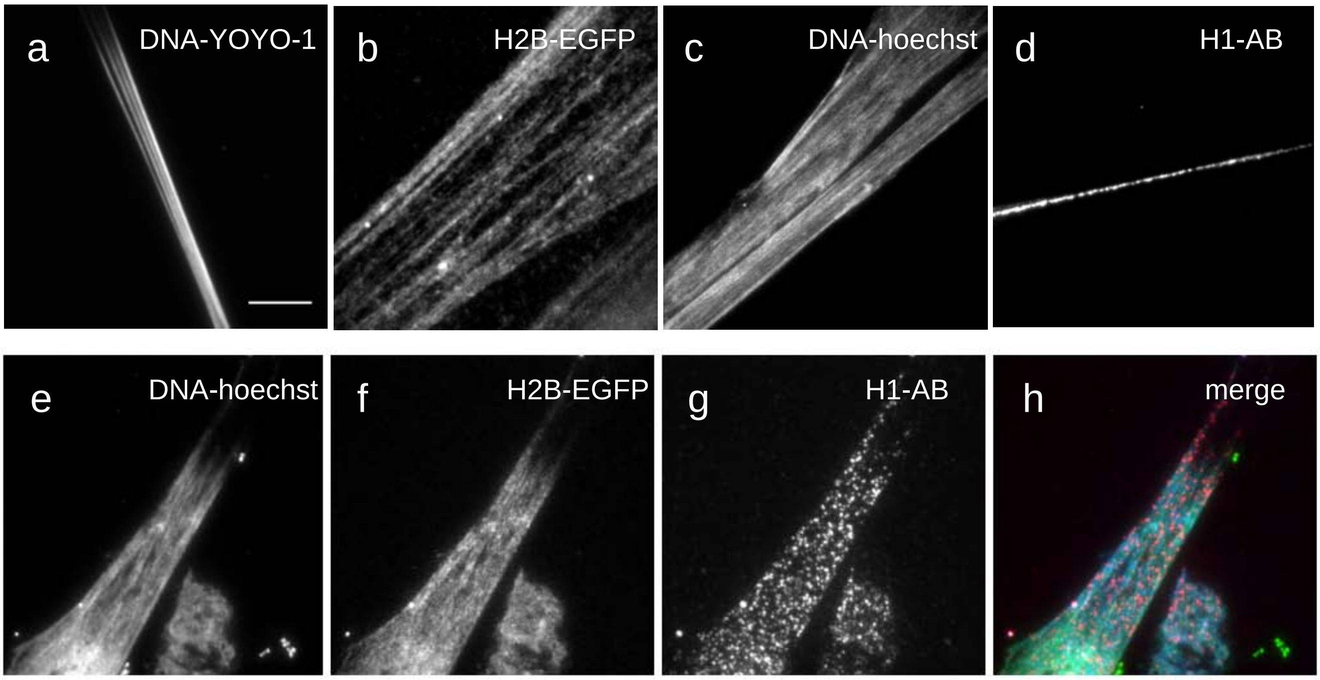

Supplementary Fig S1. Various chromatin spreads with four labeling methods and the structural integrity of spreads. | (a) DNA stained with YOYO-1. (b) Histone H2B tagged with EGFP. (c) DNA stained with heochst. (d) Histone H1 immunolabeled with its specific antibody. (e-h) Colocalization of DNA, H2B, and H1. Scale bar: 10 µm.

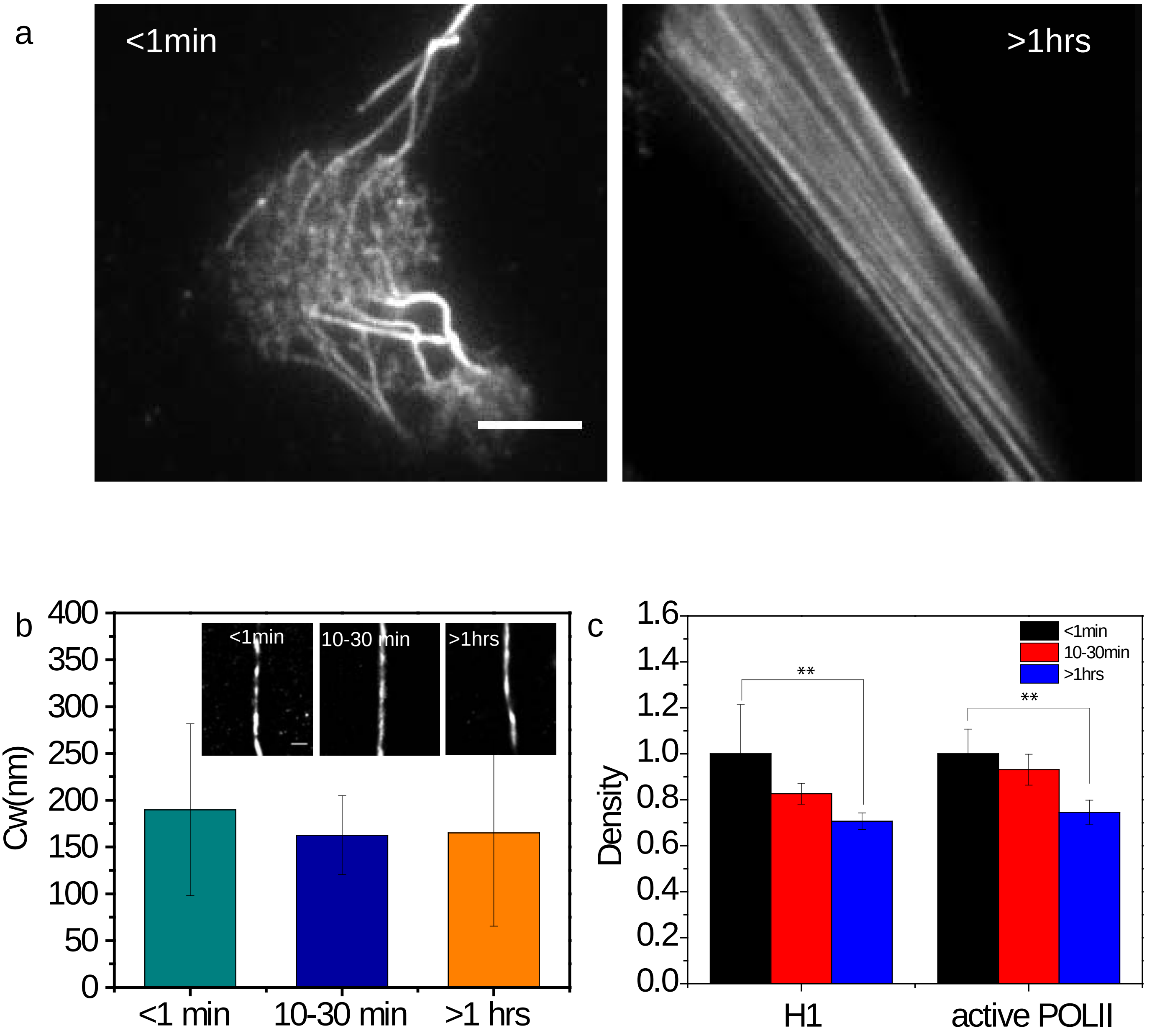

Supplementary Fig S2. Effect of expansion time on the structure of chromatin fibers and the loss of histone proteins as well as transcription machinery. | (a) TIRF images of chromaitn spreads after expasion for <1 min, >1 hrs. Scale bar: 5 μm. (b) Box graph shows the chromatin width after expansion for <1 min , 10-30 min, and >1 hrs .Inset: representative BALM images of chromatin fibers in the three expansion time. Scale bar: 500 nm. (c) Bar graph shows the density of histone proteins and RAN pol II in the three expansion time (n≥20) (***P<0.001; Student's t-test).

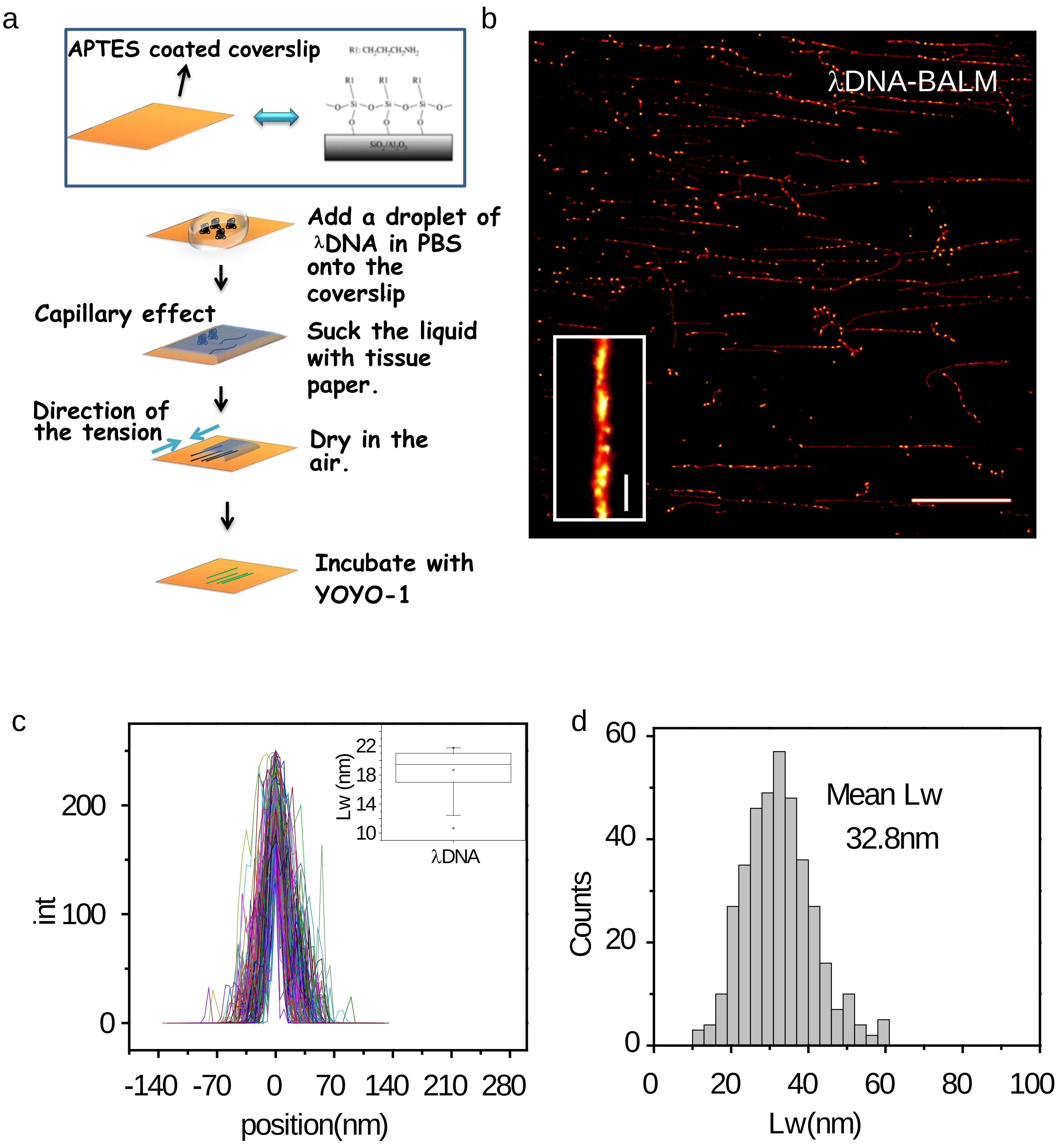

Supplementary Fig S3. Characterization of λDNA in BALM | (a) Schematic of λDNA combing (b)Representative BALM image of λDNA. Scale bar: 10 μm. Inset: zoomed in BALM image of λDNA. Scale bar: 50 nm. (c) Intensity line profiles at different regions of λDNA in BALM. Inset: Box plot of the widths of the thinnest λDNA as shown in inset of (b). (d) Histogram of lambda DNA width (Lw).

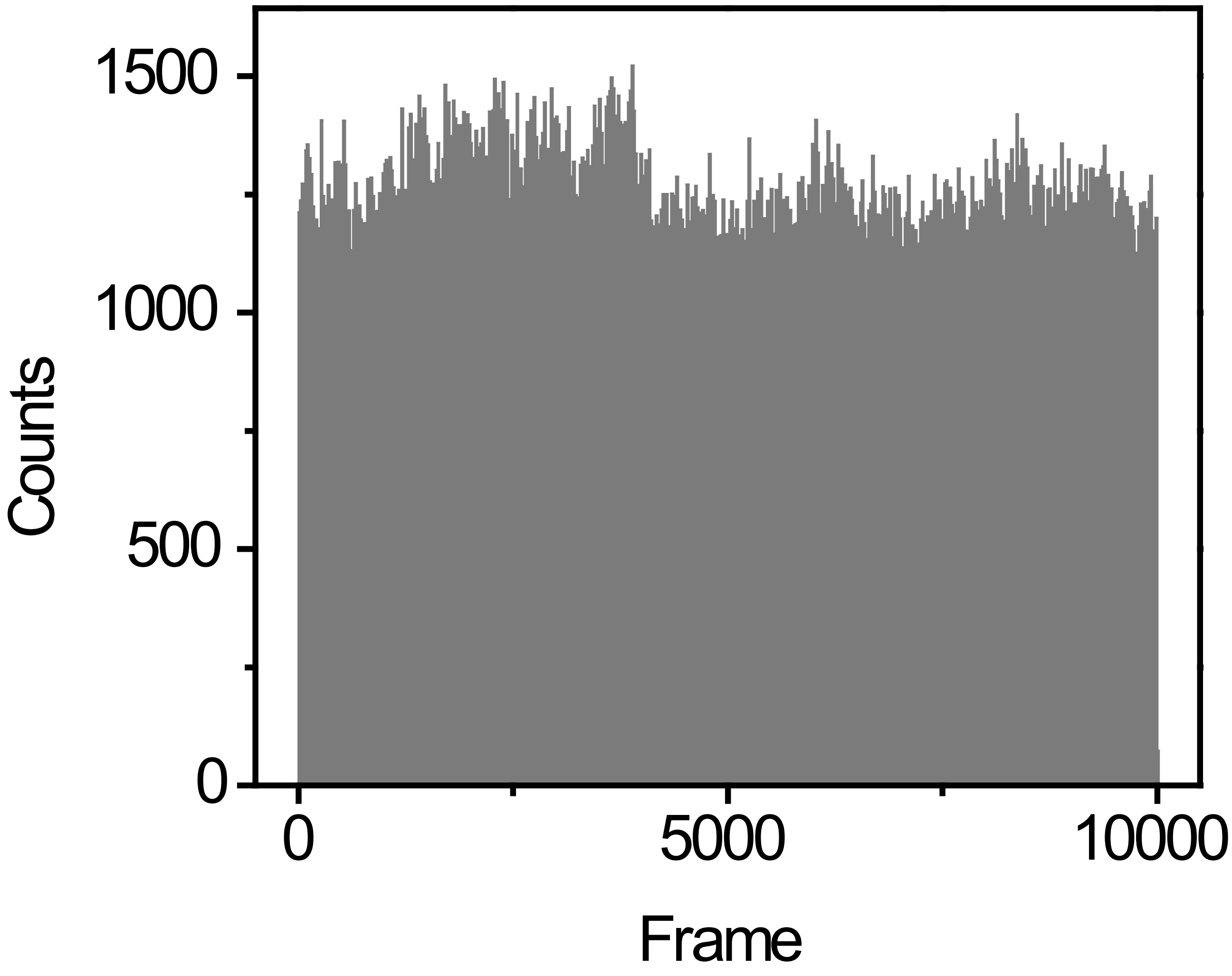

Supplementary Fig S4.Number of localization events at each of the ten thousand frames.

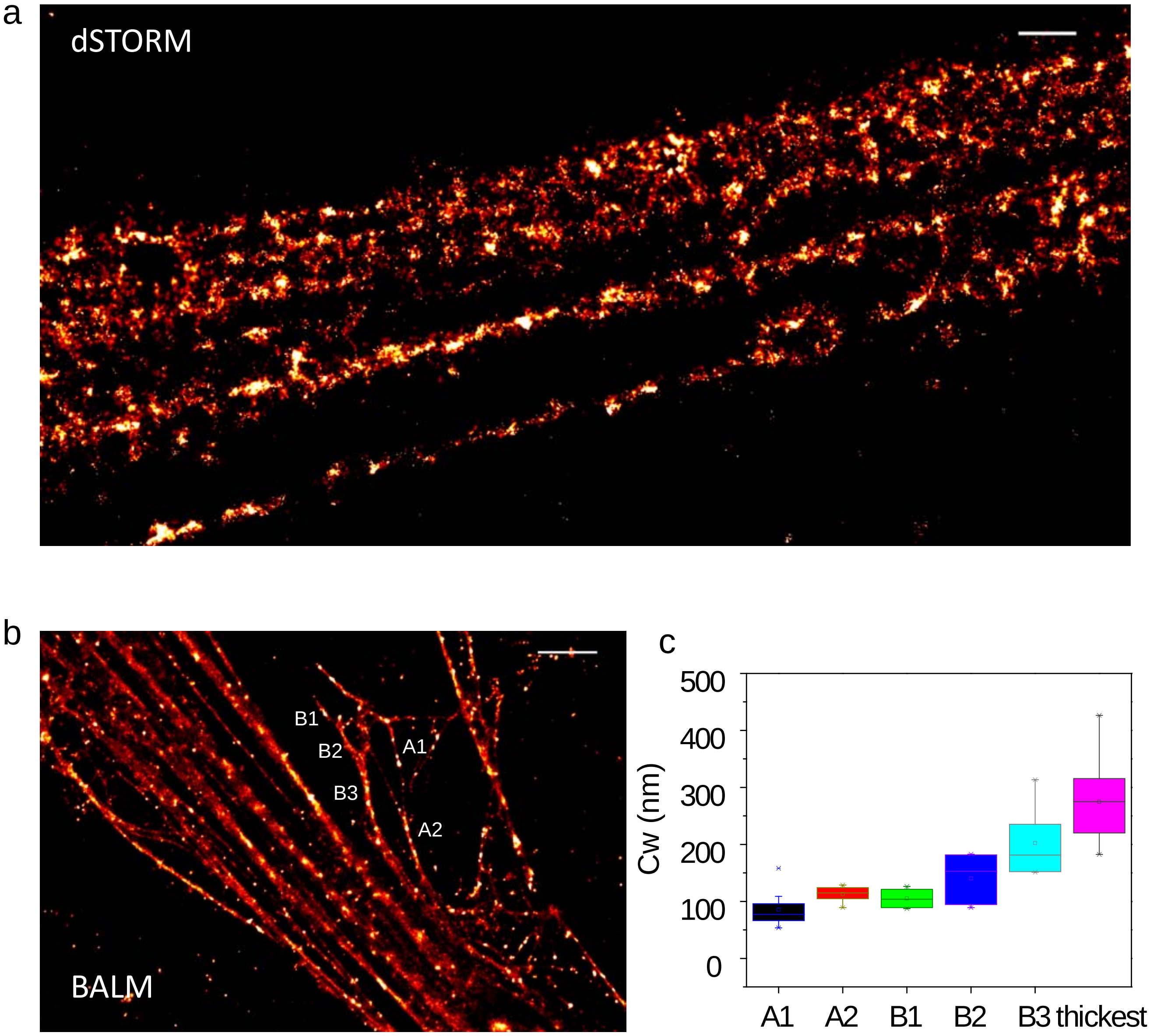

Supplementary Fig S5. Chromatin fibers detected in PALM and BALM | (a) Representative PALM image of chromaitn spreads. Scale bar: 2 μm. (b) Representative BALM image of chromatin spreads. Scale bar: 2 μm. (c) Box graph of chromaitn width (Cw) at different regions marked in (b).

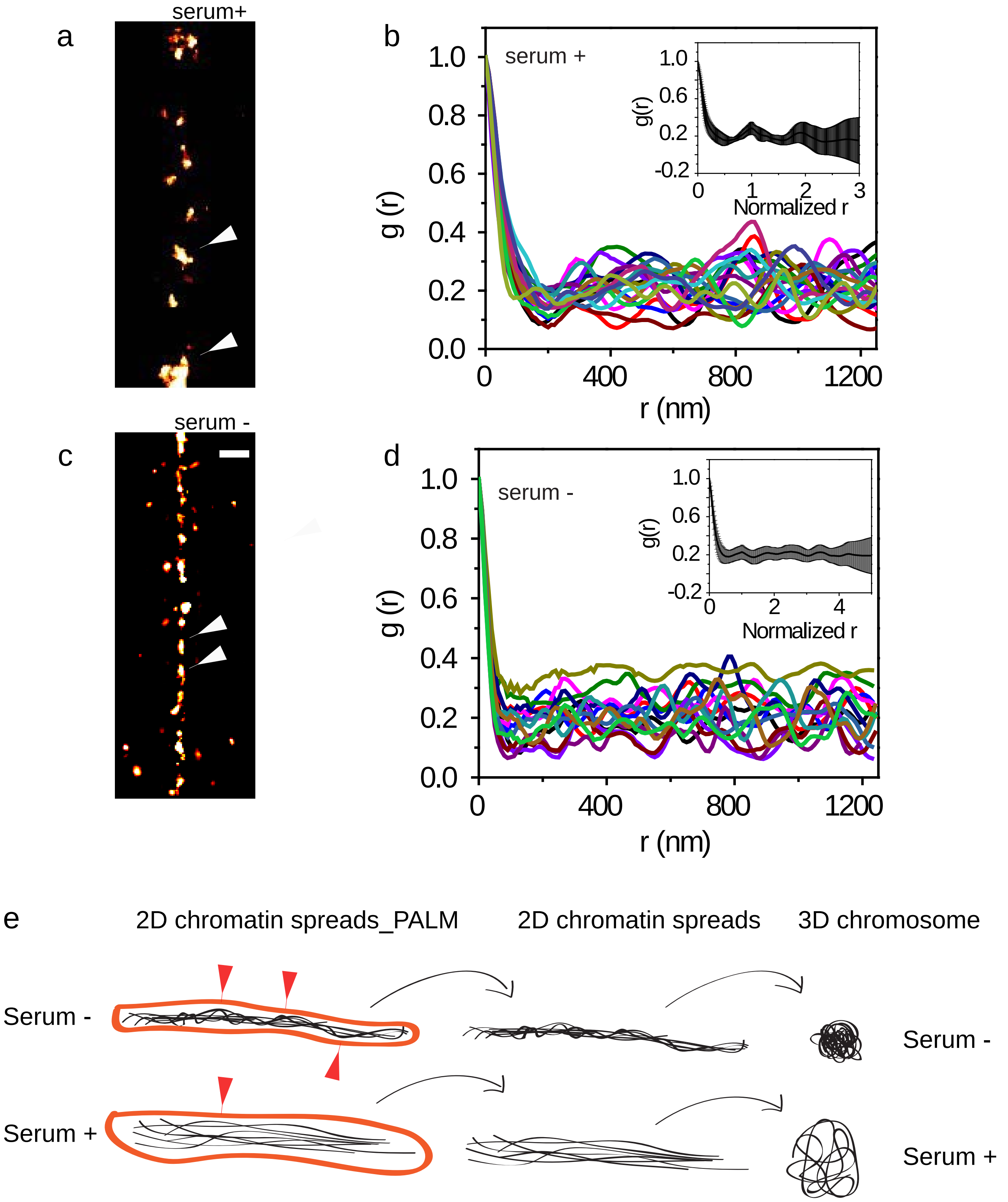

Supplementary Fig S6. Spatial correlation analysis of chromatin fibers in serum -/+ conditions | (a) Filtered image of super-resolution serum + chromatin spread. White arrow heads indicate the nodes. (b) Spatial correlation of multiple serum + chromatin fibers. Inset: averaged spatial correlation of multiple serum + chromatin fibers. (c) Filtered image of super-resolution serum - chromatin spread. White arrow heads indicate the nodes. Scale bar: 200 nm. (d) Spatial correlation of multiple serum - chromatin fibers. Inset: averaged spatial correlation of multiple serum - chromatin fibers. (e) Possible model of chromatin structure in serum -/+ conditions. Red arrow heads indicate the node structures.

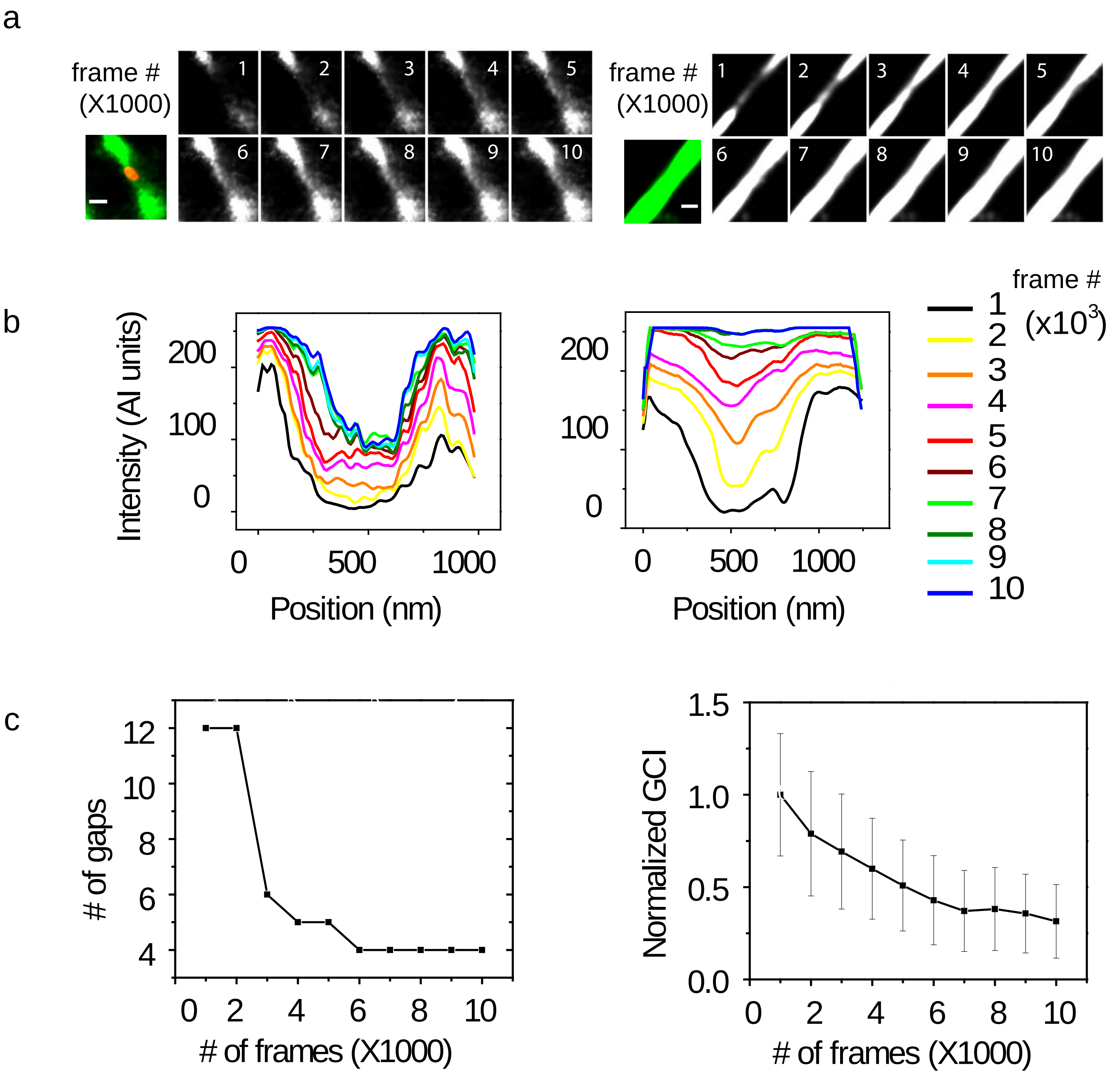

Supplementary Fig S7. Transcriptionally active gap structures are independent of frame numbers or photons collected while imaging. | (a) Collage of BALM images of chromatin fiber with different frame numbers. The cluster in red is RNA pol II. Scale bar: 200 nm. (b) Intensity line profiles showing that the active gap still remained, while the inactive gap was filled up with the increase in the frame number. (c) The graphs showing that the number of gaps as well as the normalized gaps length (GCl) decrease as the frame number goes up and finally became almost constant after ~7000 frames.

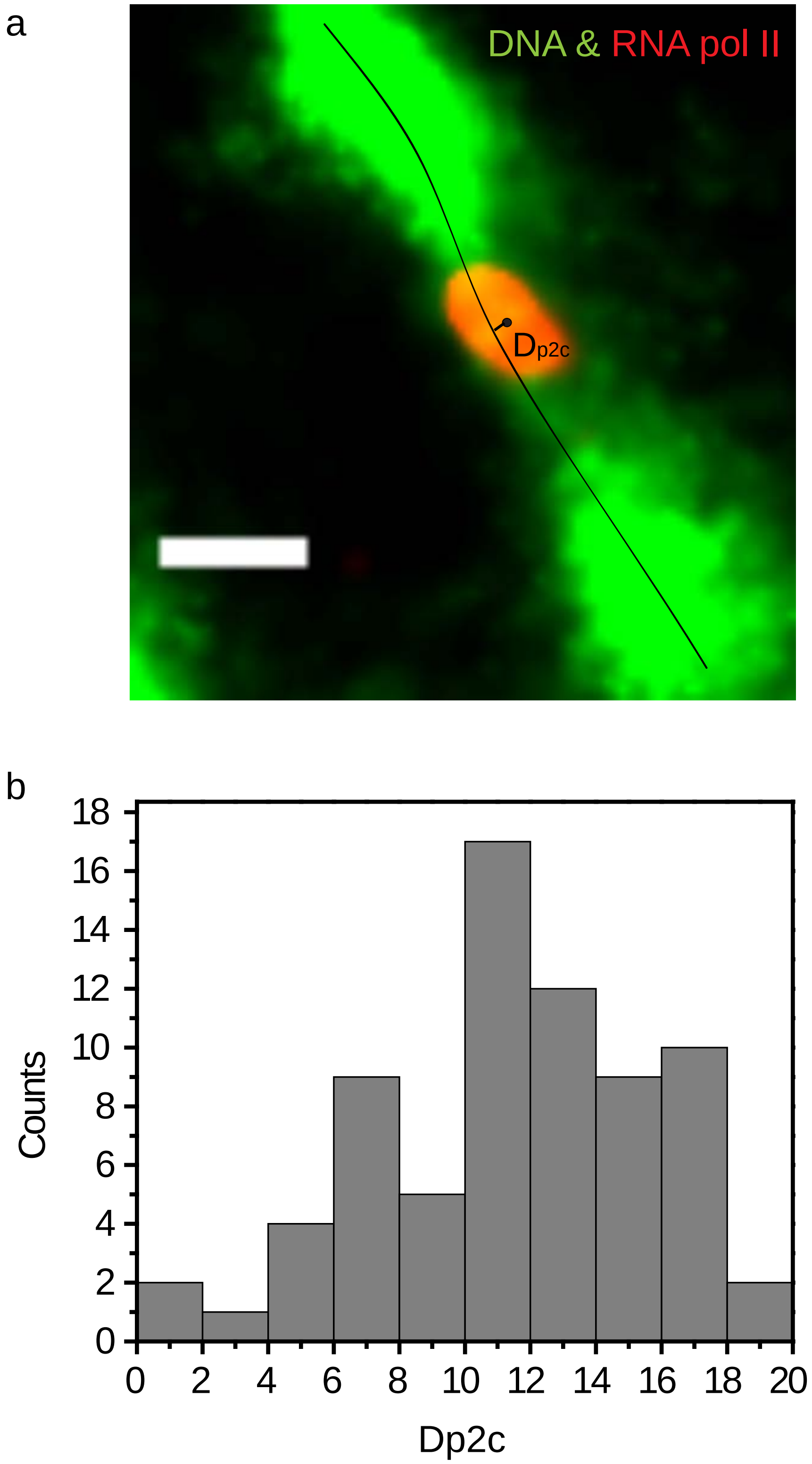

Supplementary Fig S8. Colocalization analysis for gap structures and RNA pol II | (a) A representative dual-color BALM image of gap structures (green) and RNA pol II (red). Scale bar: 200 nm. The distance between the centroid of RNA pol II staining and the cross-section center of gap (D_{p2c}) is denoted in black on the image. (b) Histogram of D_{p2c} shows that the selected 72 gap strucutres enriched with RNA pol II have D_{p2c} within 20 nm.

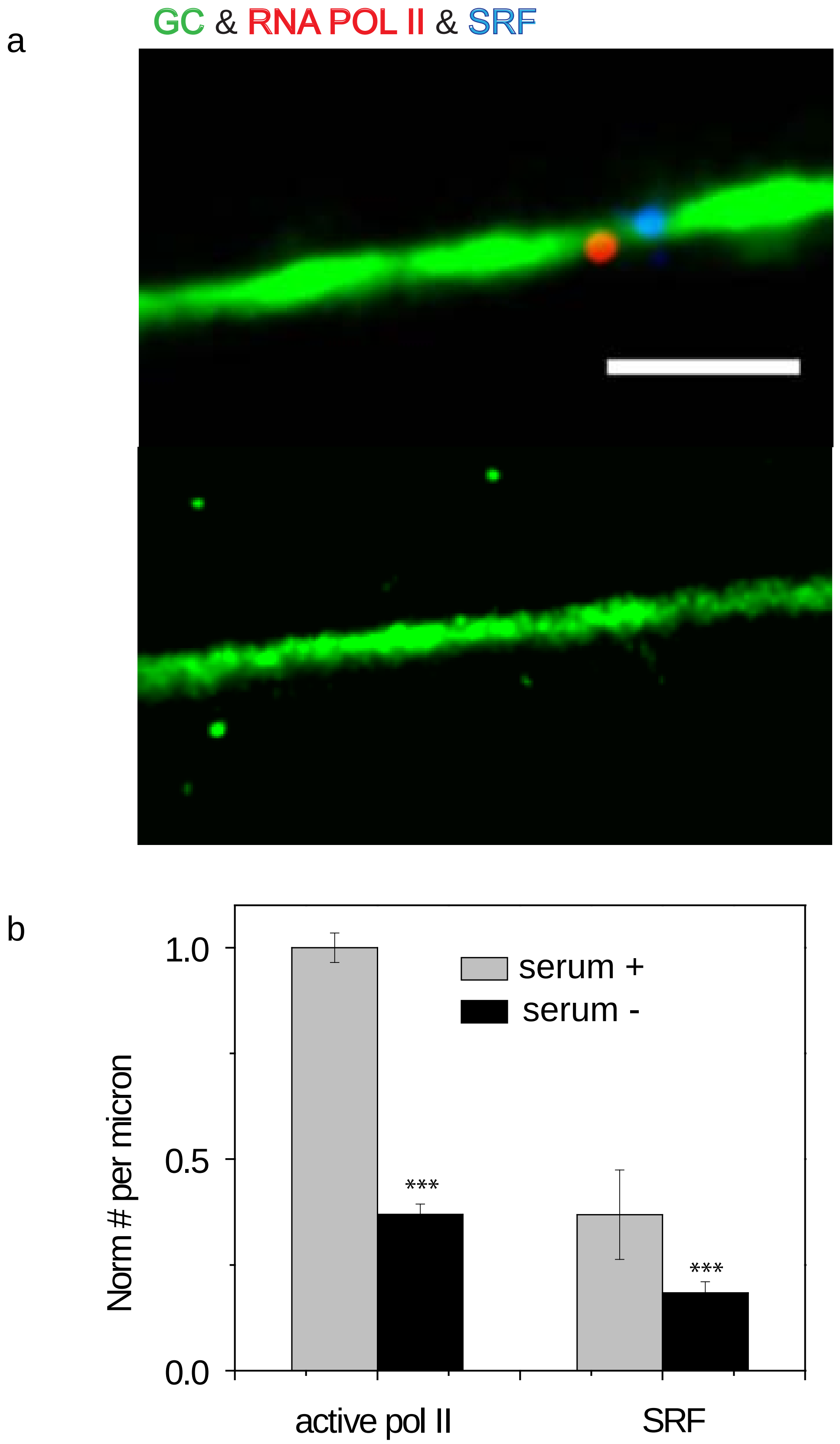

Supplementary Fig S9. The presence of active pol II and SRF at serum+/- conditions | (A) Representative three-color BALM images of chromatin (green), active pol II (red) and SRF (blue) in serum +/- conditions. Scale bar: 500 nm. (B) Bar graph of the density of active pol II and SRF along chromatin fibers in serum +/- conditions (n≥10) (***P<0.001; Student's t-test).

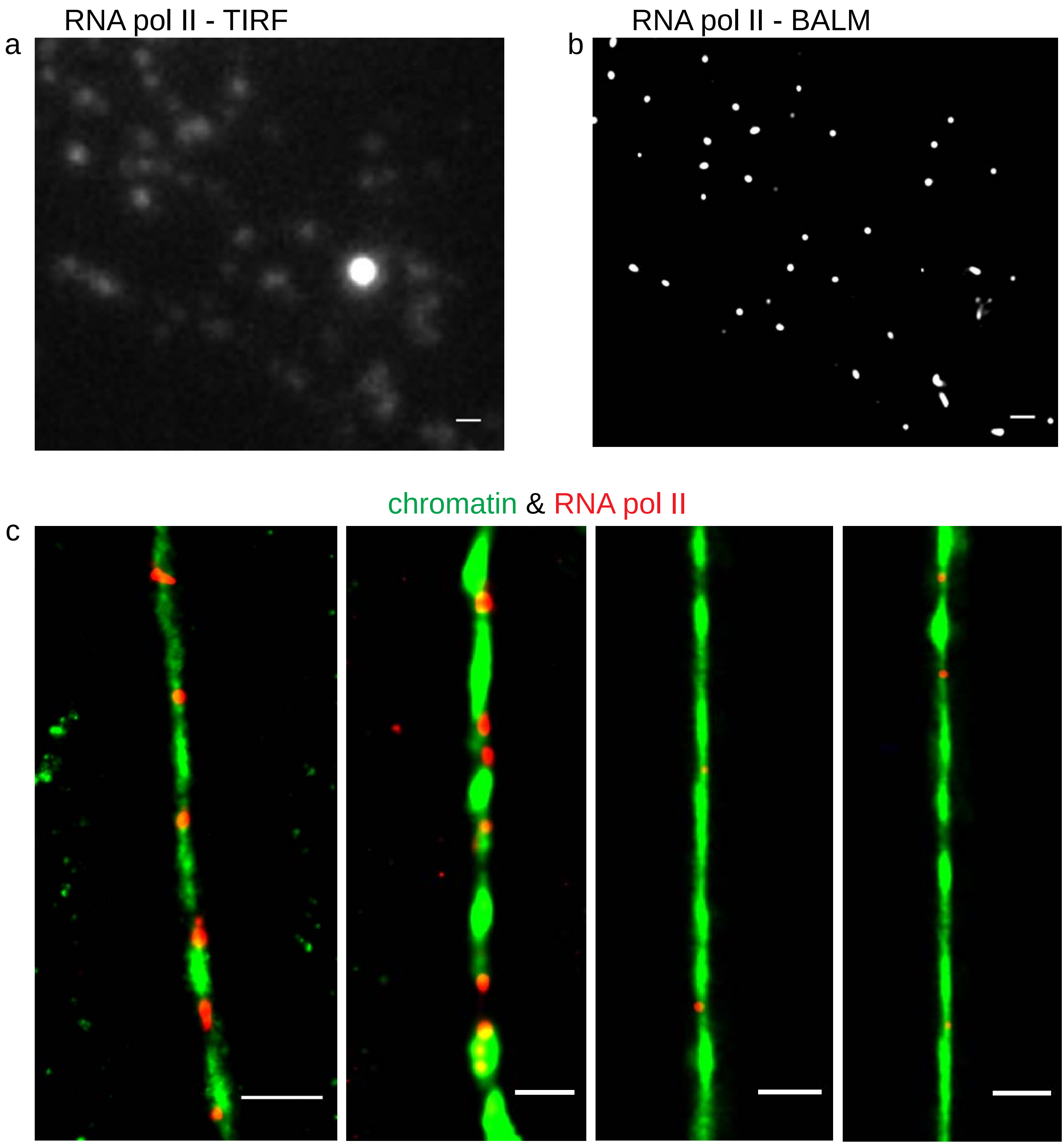

Supplementary Fig 10. Super-resolution images of RNA pol II | (a) Representative TIRF image of RNA pol II. (b) Representative BALM image of RNA pol II. Scale bar: 500 nm. (c) Representative dual-color BALM images of chromatin fibers (green) and RNA pol II (red). Scale bar: 500 nm.

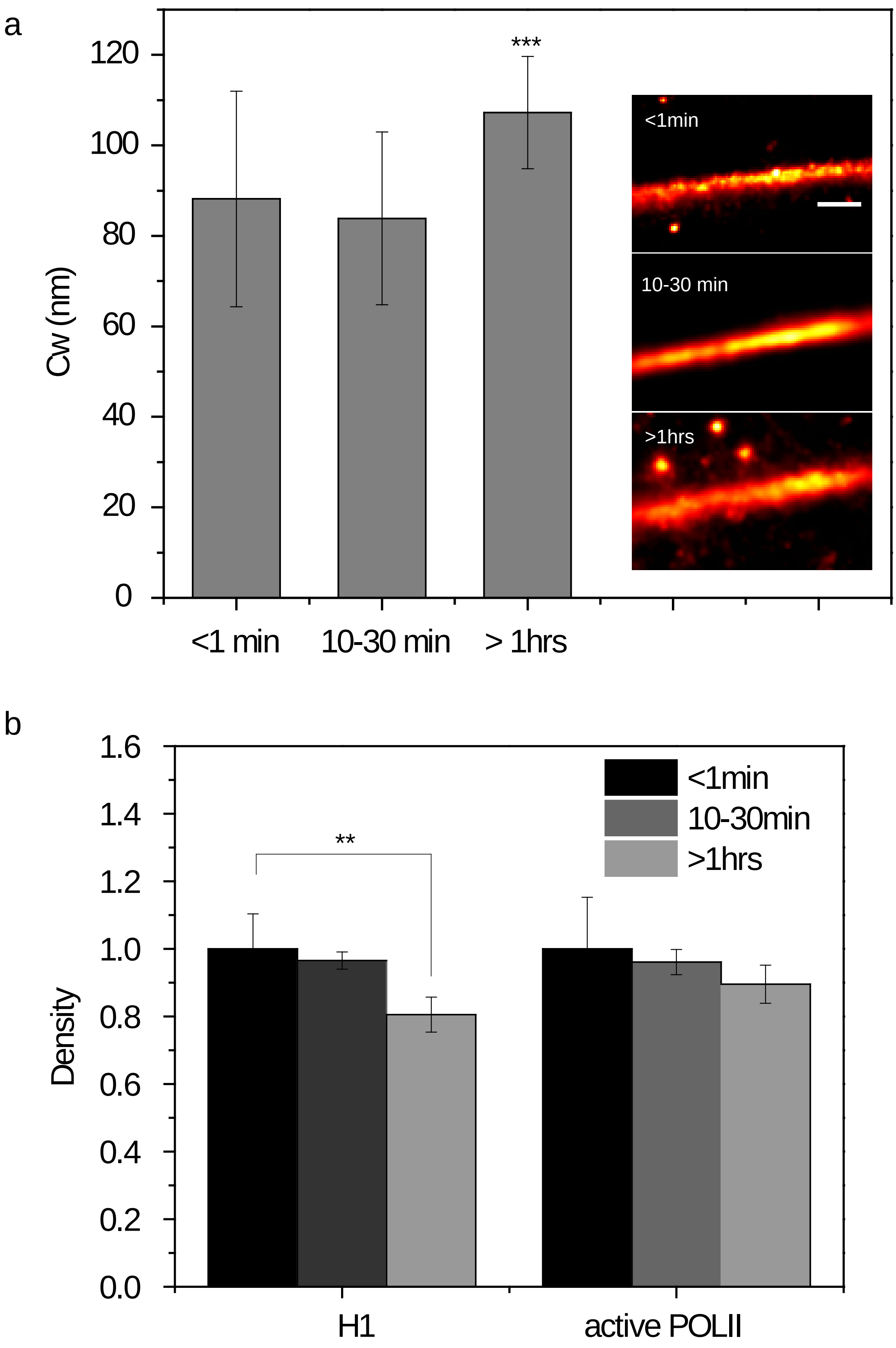

Supplementary Fig S11. Expansion sensitivity of serum-starved chromatin fibers| (a) Box graph shows the chromatin width after expansion for <1 min , 10-30 min, and >1 hrs (n≥20, ***P<0.001; Student's t-test). Inset: representative BALM images of serum-starved chromatin fibers in the three expansion time . Scale bar: 200 nm. (b) Bar graph shows the density of histone proteins H1 and RAN pol II in the three expansion time (n≥20, **P<0.01; Student's t-test).

Supplementary Table S1. Chromatin staining methods

Chromatin staining methods	Chromatin width (nm)	Probe binding site
DNA-Heochst	460±80	The minor groove of double-stranded DNA
DNA-YOYO-1	450±30	Intercalating in double-stranded DNA
H2B-EGFP	400±50	Tagged to core histone H2B
H1-AB	500±80	Immunostained to linker histone H1
